# Expression patterns of miR-221, miR-214, and miR-375 in odontogenic keratocysts versus dentigerous cysts: an in vitro comparison

**DOI:** 10.1186/s12903-025-07289-0

**Published:** 2025-12-12

**Authors:** Sareh Farhadi, Maliheh Entezari, Dorsa Abdi, Salar Mirzaei, Sina Noruozian Fard, Sana Baghizadeh

**Affiliations:** 1 Department of Oral & Maxillofacial Pathology, Faculty of Dentistry, Tehran Medical Sciences, Islamic Azad University, Tehran, Iran; 2 Farhikhtegan Medical Convergence sciences Research Center, Farhikhtegan Hospital, Tehran Medical sciences, Islamic Azad University, Tehran, Iran; 3 Faculty of Dentistry, Tehran Medical Sciences, Islamic Azad University, Tehran, Iran

**Keywords:** Odontogenic keratocyst, Dentigerous cyst, MicroRNA, MiR-221, MiR-214, MiR-375

## Abstract

**Background:**

The early diagnosis of odontogenic cysts is crucial to prevent future complications and to choose an effective treatment plan. Dentigerous cysts (DCs) and odontogenic keratocysts (OKCs) are two common developmental cysts that affect the jaw. OKCs show locally aggressive behavior and a higher recurrence rate compared with DCs, emphasizing the need for reliable molecular diagnostic markers. MicroRNAs (miRNAs) are abnormally expressed in tumors and lesions with aggressive behavior and have potential as adjunctive biomarkers. This study investigates the diagnostic potential of miR-221, miR-214, and miR-375 expression in OKCs and DCs, due to the invasive nature of OKCs compared with DCs.

**Materials and methods:**

This study analyzed 36 paraffin-embedded odontogenic cyst tissue samples (23 OKC and 13 DC) collected from the oral pathology archive of Islamic Azad University. Total RNA, including miRNAs, was extracted using TRIzol-based protocols with RNase-free conditions. RNA purity and integrity were assessed via spectrophotometry and agarose gel electrophoresis. Reverse transcription was performed using the M-MLV RNase H kit. Specific primers for miR-221, miR-214, and miR-375 were designed and verified. Quantitative real-time PCR was conducted using SYBR Green, and delta cycle threshold (ΔCt) values were calculated.

**Results:**

miR-221, miR-214, and miR-375 showed significantly lower ΔCt values in OKCs compared with DCs (*p* < 0.001). The mean ΔCt values for miR-221, miR-214, and miR-375 in OKCs were 11.27, 10.10, and 8.18, respectively, compared with 12.95, 12.51, and 11.05 in DCs.

**Conclusion:**

The ΔCt values for miR-221, miR-214, and miR-375s were significantly lower in OKC samples, indicating higher expression levels compared with DCs. The significant upregulation of these miRNAs in OKCs compared with DCs suggests that these miRNAs may indicate predictive roles and could serve as supportive biomarkers.

**Supplementary Information:**

The online version contains supplementary material available at 10.1186/s12903-025-07289-0.

## Introduction

Odontogenic cysts and jaw osseous lesions are not generally encountered in the routine practice of many dentists. However, they can lead to bony destruction, resorption, or displacement of adjacent teeth, and even dysplasia or malignant transformation, posing challenges in diagnosis and treatment [1]. Dentigerous cysts (DCs) and odontogenic keratocysts (OKCs) are two common developmental cysts that affect the jaw. DCs are related to impacted teeth and represent the second most frequent jaw cysts; they represent 14%−20% of all jaw cysts [2, 3]. DCs are mostly asymptomatic and are typically discovered incidentally during the evaluation of unerupted teeth [4]. When symptoms do occur, they usually include pain and bone expansion. They are frequently located near the lower third molars, upper canines, lower premolars, and upper third molars. These cysts may cause delayed tooth eruption, missing teeth, or abnormal tooth positioning within the dental arch [5, 6]. In the absence of treatment, the progression of the cyst remains localized, with destruction and expansion limited to peripheral structures. Malignant transformation is extremely rare [7]. OKCs are benign lesions in the jaw of odontogenic origin and make up 10% of all jaw cysts. About 75% of these cysts occur in the mandible, mainly in the molar and ramus regions [7]. In the 2005 WHO classification, OKCs were labeled as keratocystic odontogenic tumors (KOTs) because of their aggressive behavior and relatively high recurrence rate [8]. However, the evidence for neoplastic activity was insufficient to maintain their tumor classification, and the term “KOT” was changed back to its original name, “OKC” [9]. Long-standing OKCs can potentially undergo malignant transformation, especially due to persistent chronic inflammation. Epithelial dysplasia is a diagnostic challenge because of variability in its detection and grading by different examiners and even by the same examiner at different times. The development of squamous cell carcinoma (SCC) from an OKC is not well understood. Early detection of dysplasia is crucial for effective treatment and a better prognosis [9, 10].

The family of microRNAs (miRNAs) are a group of small, conserved, non-coding RNA molecules that are approximately 18 to 24 nucleotides long. They are involved in the post-transcriptional regulation of genes [11–13]. More than 60% of all human genes are predicted to be regulated by these molecules [14].The expression of these genes often becomes dysregulated across various cancer types, with functional differences depending on the tissue type [15, 16]. In addition to their role in cancer progression and development, several miRNAs have also been implicated in clinical features such as local aggressiveness and recurrence [17, 18], which may be particularly relevant to the biological behavior of odontogenic cysts. Among the deregulated miRNAs, miR-221 is known for its oncogenic role and potential as a biomarker, as it suppresses tumor suppressor genes [19]. The family of miR-375 is also frequently downregulated in several cancer types, acting as a tumor suppressor and inhibiting malignant cell properties [20]. The family of miR-214 is another tumor suppressor miRNA that is essential for regulating tumor invasiveness, proliferation, metastasis, angiogenesis, and resistance to chemotherapy [21].

Studies have shown that OKCs and DCs can potentially be premalignant lesions [7]. Despite the importance of miRNA in the development and progression of cancers [15], their role in epithelial odontogenic cysts and tumors is not well documented. Considering the characteristic behavior of OKC in comparison to other odontogenic cysts and the high prevalence of DC [7, 9], the present study aims to compare the expression of miR-221, miR-214, and miR-375 in DCs and OKCs. To the best of our knowledge, this topic has not been previously investigated in odontogenic cysts, whereas previous research has been limited to head and neck cancers, predominantly SCC [22–25].

## Materials and methods

This in vitro study was approved by the Institutional Ethics Committee (IR.IAU.DENTAL.REC.1401.033). A total of 36 formalin-fixed paraffin-embedded (FFPE) tissue samples, including 23 OKCs and 13 DCs, were retrieved from the oral pathology archive of the Islamic Azad University, Tehran Medical Branch. All cases were histopathologically confirmed.

### RNA extraction and quality assessment

Total RNA, including miRNAs, was extracted from FFPE sections using a TRIzol-based protocol (Parstous, Iran). The RNA concentration and purity were determined with a NanoDrop spectrophotometer (Thermo Scientific, USA), and the integrity was confirmed by agarose gel electrophoresis. Samples with acceptable purity ratios were included in downstream analyses [26, 27].

### Reverse transcription

Complementary DNA (cDNA) was synthesized from total RNA using the M-MLV RNase H Kit (BioFact, Daejeon, Korea), according to the manufacturer’s instructions [28].

### Primer design

Specific primers for miR-221, miR-214, and miR-375 were designed and validated (Table 1). cDNA synthesis was performed using 1000 ng/µL total RNA per reaction, and expression data were normalized to U6 (RNU6B).

**Table 1:** Sequences of the primers

microRNA symbol	Primer Sequence
miR-221	Forward5′-ACUGGCAUACAAUGUAGAUUU-3′ Reverse5′-AGCUACAUUGUCUGCUGGGUUUC-3′
miR-214	forward 5’-TGCGGACAGCAGGCACAGAC-3’ Reverse 5’-CCAGTGCAGGGTCCGAGGT-3’
miR-375	forward 5’-GCCCGCTTTGTTCGTTCGGCT-3’ Reverse 5’-GTGCAGGGTCCGAGGT-3’

### Quantitative real-time PCR

Quantitative PCR (qPCR) was performed using SYBR Green dye (TaKaRa, Japan) on a standard thermocycler platform. All reactions were run in triplicate, and the specificity was verified by melt-curve analysis [28–30].

### Data analysis

Relative expression levels of miRNAs were calculated using the 2^-ΔΔCt method [31]. Statistical analyses were performed with SPSS software (version 29.0; IBM Corp., Armonk, NY, USA) separately for each of the three miRNAs. Data distribution was assessed using the Kolmogorov–Smirnov test, and group comparisons were conducted with independent-sample t-tests or the Mann–Whitney U test, as appropriate. A p-value < 0.05 was considered statistically significant.

## Results

In total, 36 FFPE tissue samples were analyzed, including 23 OKCs and 13 DCs. QPCR was performed to evaluate the expression of miR-221, miR-214, and miR-375. The data are presented as mean ± standard deviation (SD).

The independent t-test revealed statistically significant differences in the ΔCt values of all three miRNAs between OKC and DC groups (*p* < 0.001 for each comparison). As ΔCt values are inversely related to expression, lower ΔCt values indicate higher miRNA expression relative to the reference gene. As shown in Table 2, the mean ΔCt value for miR-221 in OKCs was 11.27 ± 0.91, which was significantly lower than 12.95 ± 0.66 in DCs (*p* < 0.001). Similarly, miR-214 showed a mean ΔCt of 10.10 ± 0.61 in OKCs versus 12.51 ± 0.46 in DCs (*p* < 0.001; Table 3), and miR-375 had a mean ΔCt of 8.18 ± 0.63 in OKCs compared with 11.05 ± 0.86 in DCs (*p* < 0.001; Table 4). Taken together, these results demonstrate that all three miRNAs were significantly upregulated in OKCs compared with DCs.

**Table 2:** Expression differences of *miR-221* between odontogenic keratocyst (OKC) and dentigerous cyst (DC) groups based on ΔCt values

	Mean ΔCT	Standard deviation	lowest	highest	*P* value
OKC	11.2691	0.90787	9.87	12.93	< 0.001
DC	12.9523	0.65736	11.69	14.09	< 0.001

**Table 3:** Expression differences of *miR-214* between odontogenic keratocyst (OKC) and dentigerous cyst (DC) groups based on ΔCt values

	Mean ΔCT	Standard deviation	lowest	highest	*P* value
OKC	10.0978	0.60916	9.39	11.42	< 0.001
DC	12.5091	0.45686	11.89	13.34	< 0.001

**Table 4:** Expression differences of *miR-375* between odontogenic keratocyst (OKC) and dentigerous cyst (DC) groups based on ΔCt values

	Mean ΔCT	Standard deviation	lowest	highest	*P* value
OKC	8.1803	0.63364	6.83	9.69	< 0.001
DC	11.0542	0.86091	9.80	12.54	< 0.001

**Fig. 1:**
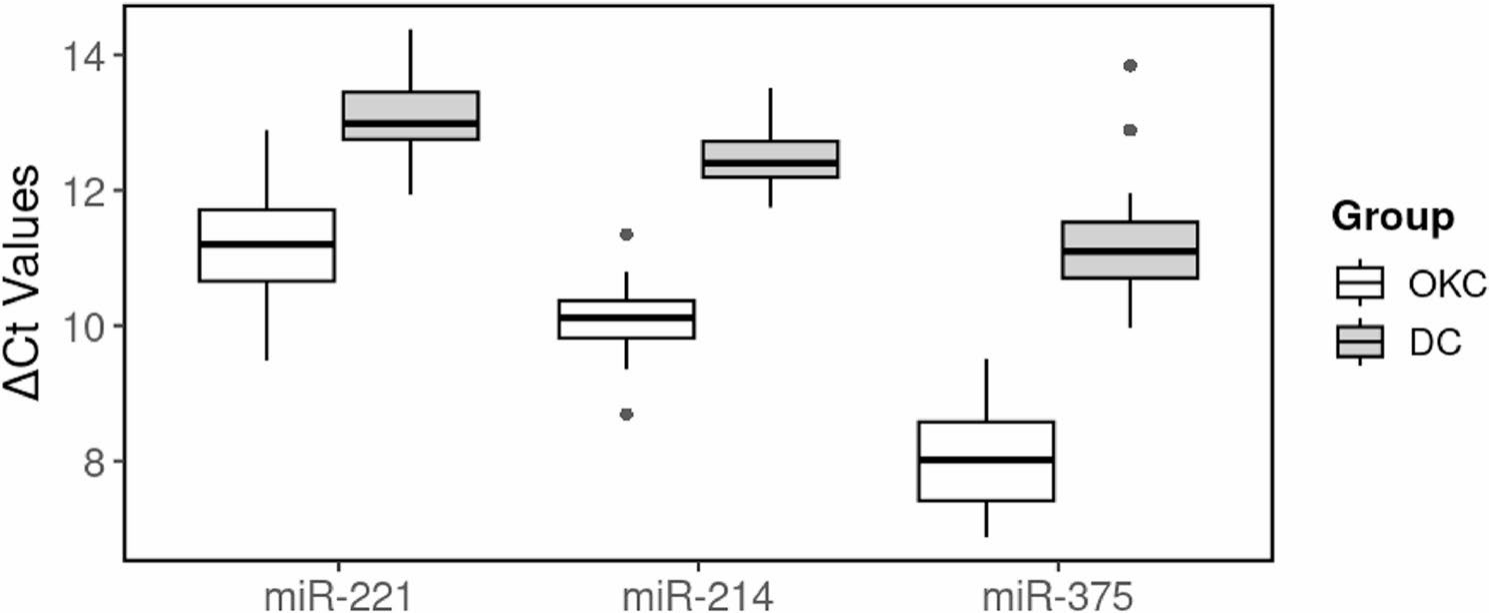
Expression differences of miRNAs between odontogenic keratocyst (OKC) and dentigerous cyst (DC) based on ΔCt values

These ΔCt differences were further translated into relative expression values and fold changes using the 2^(-ΔΔCt) method. As summarized in Table 5, the fold change of miR-221, miR-214, and miR-375 in OKC tissues was markedly higher than in DC tissues. Figure 1 graphically illustrates the differences in ΔCt values between the two groups.

**Table 5:** The calculated relative expression levels and fold changes (Odontogenic keratocyst vs. dentigerous cyst)

	Relative Expression in OKC	Relative Expression in DC	Fold Change (OKC/DC)
miR-221	0.000405	0.000126	3.20
miR-214	0.000911	0.000171	5.31
miR-375	0.003448	0.000472	7.31

## Discussion

We investigated the Expression patterns of miR-221, miR-214, and miR-375 in OKCs and DCs, as these tumors exhibit aggressive growth and higher recurrence rates compared with DCs. Our results demonstrate that the expression levels of all three miRNAs in OKCs are significantly elevated in OKC samples relative to DCs. This differential expression suggests that these miRNAs may provide adjunctive information in differentiating OKCs from DCs, although histopathology remains the gold standard. Furthermore, upregulation of certain miRNAs in OKCs may reflect underlying molecular pathways associated with the more proliferative and invasive nature of this lesion. To the best of our knowledge, this is the first study to investigate the expression of these specific miRNAs in odontogenic cysts, highlighting novelty and potential contribution to the field.

Epithelial odontogenic cysts and tumors originate from the odontogenic epithelium and the surface epithelial lining of the oral mucosa. The difference in the proliferation of epithelial cells plays an important role in the pathogenesis of these lesions [32]. The biological behavior of a lesion is generally associated with its growth potential, which can be assessed by measuring cellular proliferative activity. This growth potential, and its correlation with clinical aggressiveness and recurrence, can help determine the lesion’s nature. Experimental studies indicate that the extent of cellular proliferation within a lesion can serve as an indicator of its biological aggression [33]. miRNAs are important regulators of gene expression and have been implicated in modulating cellular proliferation, apoptosis, and other processes associated with the behavior of tumors. Altered expression of these miRNAs has been implicated in early phases of cancer development; however, in the context of odontogenic cysts, their role remains unclear. Several studies have reported that most of the identified miRNA genes are located within cancer-associated genomic regions, often referred to as fragile sites. As such, identifying these miRNAs may contribute to understanding biological behavior and could potentially support the assessment of malignant potential [25, 34–37]. Although miRNAs have been implicated in cancer development and progression, only a few studies have specifically addressed their role in clinical features such as local aggressiveness and recurrence [17, 18, 38, 39].

Understanding the role of miRNA in cancer biology is challenging because of its biphasic nature, where the same miRNA can function as an oncogene in one cancer type and as a tumor suppressor in another. For example, miR-29 functions as an oncogene in breast, bladder, colorectal, and malignant pleural mesothelioma cancers, but acts as a tumor suppressor in pancreatic, hepatocellular, gastric, and renal cell carcinomas. This dual role is attributed to miRNAs targeting different messenger RNAs (mRNAs) across various tissues, often through distinct signaling pathways [16].

According to past studies, miR-221, miR-214, and miR-375 are not only heavily dysregulated but also exhibit variable expression levels in cancer tissues [2, 40, 41]. Recent research has used miRNAs or specific miRNA ratios as prognostic markers in head and neck squamous cell carcinoma. For example, the ratio of miR-221 to miR-375 was highly specific (0.93) and sensitive (0.92) for distinguishing normal from malignant tissues [42].

The family of miRNA-221 is part of the miRNA-221/222 cluster encoded in chromosome Xp11.3 [43], and it has dual roles in cancer as an oncomiR or tumor suppressor [19, 44]. He et al. reported that miR-221 expression was elevated in Oral Squamous Cell Carcinoma (OSCC) tissues, suggesting its potential as a therapeutic target for OSCC management [45]. In another study, Yang et al. analyzed the the crosstalks between SCIRT and miR-221 and confirmed the upregulation of miR-221 in OSCC cells [46]. Interestingly, in contrast, it was found that miR-221 acted as an oncosuppressor in erythroleukemic cells [47]. Ravegnini et al. conducted a systematic review and meta-analysis of the prognostic role of miR-221 and miR-222 expression in cancer patients. They reported that the prognostic role of miR-221was inconsistent and less robust than that of miR-222, and the significance and role of miR-221 remained unclear and controversial [48].

The family of miR-214 is located on chromosome 1q24.3 [21], and it was initially identified for its ability to induce apoptosis in HeLa cells by inhibiting the anti-apoptotic protein Bcl2l2 and enhancing pro-apoptotic factors like Bax and caspases [49]. Additionally, it targets oncogenic mRNAs, including CD44 and CDK1, and leads to reduced proliferation and migration in various cancers [50]. MiR-214 exhibits variable expression across cancer types and functions as both a tumor suppressor and an oncogenic miRNA [51]. In OSCC, it induces malignancy through the MAPK/ERK pathway and correlates with poor prognosis [29], while its inhibition enhances apoptosis [52]. A miR-based prognostic model, including miR-214-3p, appeared to be effective for detecting early-stage OSCC [25]. Yang et al. demonstrated that miR-214 plays a role in oral cancer progression and that its levels are reduced following chemotherapy [53].

The family of miR-375 is located on chromosome 2 and plays tissue-specific roles as both a tumor suppressor and an onco-miRNA [54, 55]. It suppresses malignancy in nasopharyngeal carcinoma [56] but promotes breast cancer progression by targeting HOXA5 [57]. In oral potentially malignant disorders (OPMD), PCNA miR-375 levels are lower in patients with dysplasia, which indicates its potential as a sensitive marker [58]. In OSCC, downregulation of miR-375 correlates with poor prognosis, reduced survival, and lymph node metastasis [59]. Elevated miR-375 levels inhibit proliferation and invasion by targeting p53 and SLC7A11, enhancing apoptosis and radiosensitivity [60].

Based on the findings of this study and considering that specific miRNAs exhibit high tissue and organ specificity, it seems that assessing the expression of particular miRNAs could be valuable for predicting the clinical behavior of odontogenic cysts. Currently, techniques such as qRT-PCR and microarray are used for miRNA profiling, which are non-invasive, cost-effective, and reliable [61]. However, several challenges remain when using miRNAs, such as the complex nature of miRNA-mRNA interactions. A single miRNA can target multiple mRNAs and vice versa. Therefore, standardizing or normalizing miRNA levels in experimental studies of these interactions requires extensive research, which can be challenging. Fortunately, bioinformatics tools and algorithms are available to predict and assess miRNA-mRNA interactions based on sequence data [36]. Studies from various countries have reported variations in miRNA expression in oral cancer patients, raising concerns about the reliability of miRNAs as biomarkers. For example, miR-21 levels were higher in plasma from Japanese and Danish cohorts, but lower in the Turkish cohort and unchanged in Chinese patients [62]. Consequently, further studies are necessary to provide a clearer explanation.

This study has several limitations. First, the use of FFPE tissue for miRNA extraction may affect RNA quality, as tissue lysis can alter miRNA concentrations and potentially influence the outcome [63, 64]. This emphasizes the need for additional investigations utilizing fresh tissue samples. Second, an independent validation cohort was not available in our archive, which may restrict the reliability of the proposed biomarkers. Future studies should also include additional control tissues, such as hyperplastic dental follicle tissue or other odontogenic lesions, to provide a more comprehensive understanding of whether the observed miRNA expression patterns are specific to OKCs and DCs. Third, the sample size was relatively small (23 OKCs and 13 DCs), which may limit the statistical power and generalizability of the findings. In addition, Receiver Operating Characteristic (ROC) curve analysis and diagnostic performance metrics (sensitivity, specificity, AUC) were not performed due to the limited sample size and exploratory design of this study.

Despite the limitations, the differential expression of miR-221, miR-214, and miR-375 suggests their potential utility as molecular predictors of biological behavior, possible local aggression, recurrences, and malignant conversion. Their application in research and future clinical diagnostics might facilitate early detection and management of odontogenic cysts with aggressive behavior, reducing recurrence and unnecessary extensive surgical interventions. However, histopathology remains the gold standard.

## Conclusion

In summary, a significant difference in the expression levels of miR-221, miR-214, and miR-375 was observed between OKCs and DCs. These findings suggest that altered miRNA expression may reflect the more aggressive biological behavior of OKCs. However, histopathological examination remains the gold standard for diagnosis, and the role of these miRNAs should be regarded only as potential adjunctive indicators. Larger, independent studies are required to confirm their predictive value and clarify their contribution to the pathogenesis of odontogenic cysts.

## Supplementary Information

Supplementary Material 1.

Supplementary Material 2.

Supplementary Material 3.

Supplementary Material 4.

## Data Availability

The research data supporting the results of this manuscript are available from the corresponding author upon reasonable request.

## Abbreviations

DC: Dentigerous cysts
OKC: Odontogenic keratocysts
miRNAs: MicroRNAs
ΔCt: Delta cycle threshold
FFPE: Formalin-fixed paraffin-embedded
cDNA: Complementary DNA
qPCR: Quantitative PCR
SD: Standard deviation
KOTs: Keratocystic odontogenic tumors
SCC: Squamous cell carcinoma
OSCC: Oral Squamous Cell Carcinoma
OPMD: Oral potentially malignant disorders
ROC: Receiver Operating Characteristic
AUC: Area Under the Curve
