# Supplementary material for: Expression patterns of miR-221, miR-214, and miR-375 in odontogenic keratocysts versus dentigerous cysts: an in vitro comparison
Source: BMC Oral Health. 2025 Dec 12;25:1896. doi: 10.1186/s12903-025-07289-0 (PMC12699812; doi:10.1186/s12903-025-07289-0)
Supplement: Supplementary file 1 — Supplementary Material 1. [file 12903_2025_7289_MOESM1_ESM.docx]

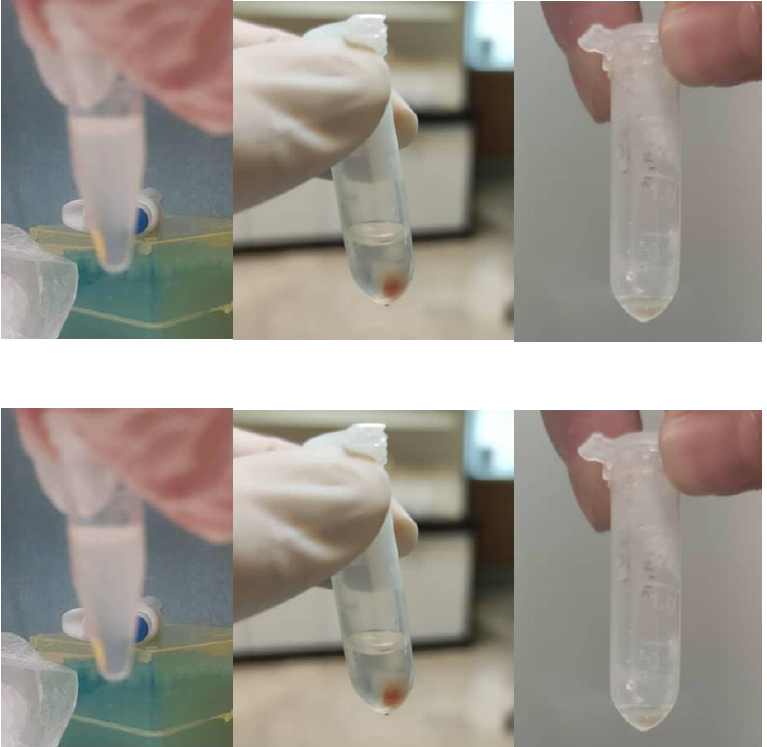


Figure 1. RNA extraction procedure – RNA from tissue in the plate and after extraction


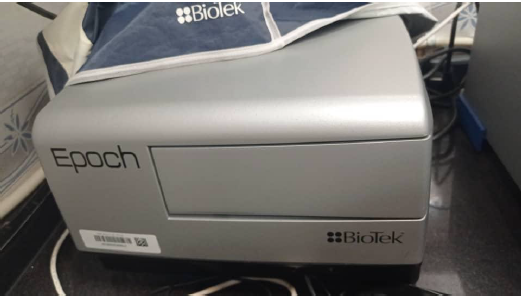


Figure 2. NanoDrop spectrophotometer


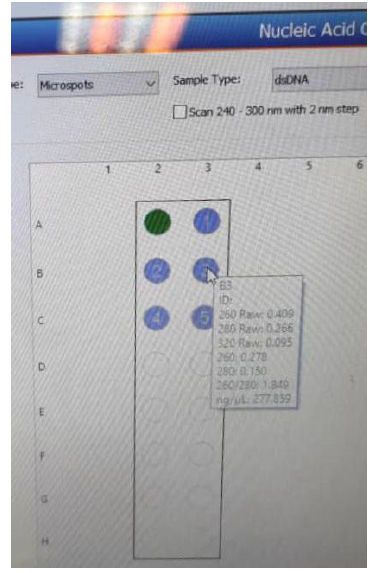


Figure 3. Concentration of extracted RNA.


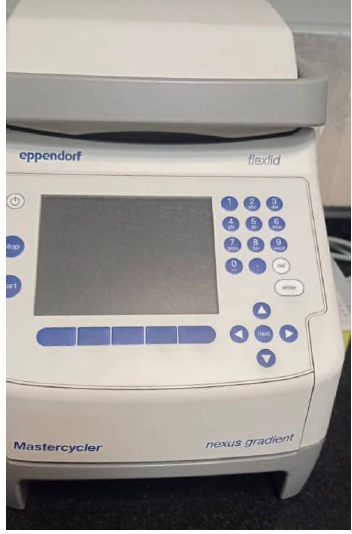


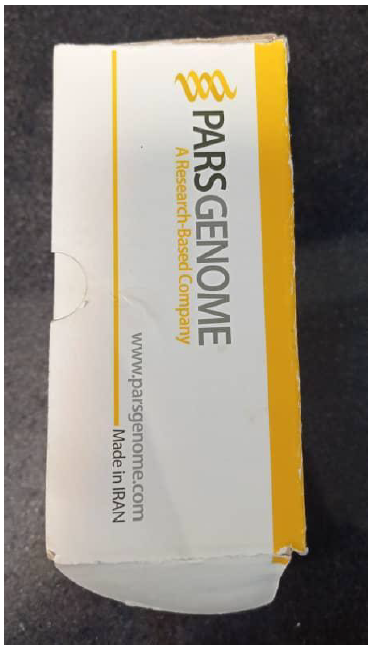


Figure 4. The real-time PCR machine

Figure 5. cDNA synthesis kit


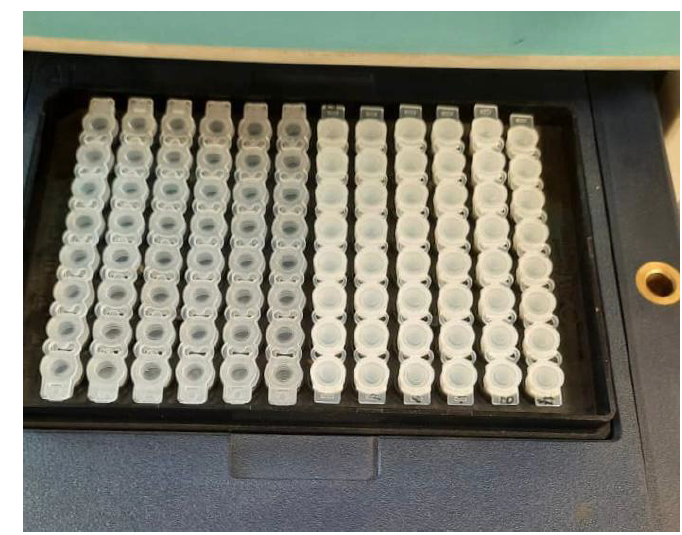


Figure 6. Samples loaded in the Real-Time PCR machine


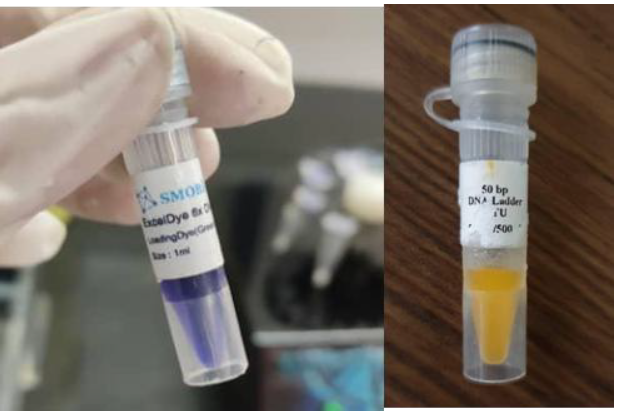


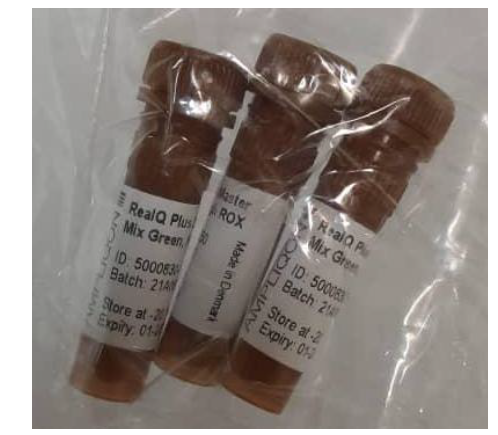
Figure 7. Dye for gel electrophoresis and 50 bp ladder

Figure 8. Syber Green


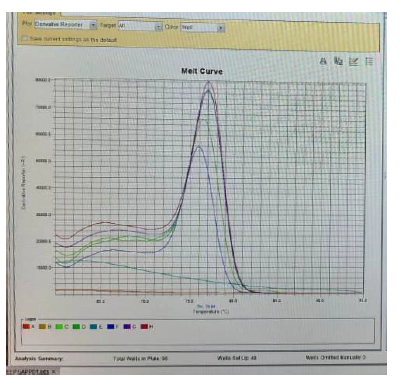


Figure 9. Melt curve plot for miR214


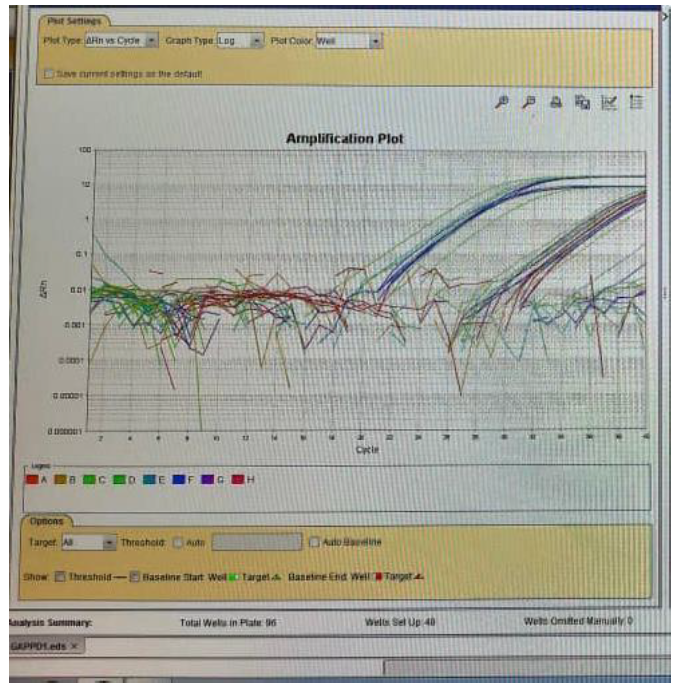


Figure 10. Amplification plot graph showing amplification; the first curve corresponds to the housekeeping gene, and the second curve corresponds to the miR214 gene
